# Identifying novel biomarkers associated with bladder cancer treatment outcomes

**DOI:** 10.3389/fonc.2023.1114203

**Published:** 2023-03-29

**Authors:** Peris R. Castaneda, Dan Theodorescu, Charles J. Rosser, Michael Ahdoot

**Affiliations:** ^1^ Department of Urology, Cedars-Sinai Medical Center, Los Angeles, CA, United States; ^2^ Samuel Oschin Comprehensive Cancer Institute, Los Angeles, CA, United States

**Keywords:** biomarker, bladder cancer, genetic testing, treatment, targeted therapy

## Abstract

Bladder cancer is a complex disease with variable prognosis. Recent investigations into the molecular landscape of bladder cancer have revealed frequent genetic alterations and molecular subtypes with therapeutic implications. Consequently, a shift toward personalized treatment of bladder cancer is underway. To this end, several biomarkers have been developed and tested in their ability to predict response to treatment in patients with bladder cancer and potentially help direct therapy. We performed a search of recently published PubMed articles using terms “biomarker,” “bladder cancer,” and the respective treatment discussed (i.e., “neoadjuvant” or “BCG”). In this review, we summarize the latest studies on novel biomarkers in bladder cancer with a focus on those intended to improve risk stratification and treatment selection.

## 1 Introduction

Bladder cancer (BC) is the most common malignancy of the urogenital tract. More than 1.6 million people are living with BC worldwide. Over 80,000 new cases and 17,000 deaths are expected in the United States alone in 2022 (1). At initial diagnosis, approximately 75% of people have non-muscle invasive bladder cancer (NMIBC), with the remainder having muscle invasive bladder cancer (MIBC) or metastatic disease (2). In addition to personal cost, BC incurs a significant healthcare cost. BC has the highest lifetime treatment costs per patient of any cancer (3). These costs are amplified by the high recurrence and progression rates of NMIBC (4).

The treatment of BC is complex and outcomes are varied. Treatments for both NMIBC and MIBC are based on clinical and histopathologic information. However, only a subset of patients derive benefit from these treatments. Robust biomarkers are needed to manage patients with both NMIBC and MIBC. In the United States, no biomarker is has gained Food and Drug Administration (FDA) approval for use in therapeutic decision making and none are widely used in clinical practice.

This situation underscores the need to develop and validate biomarkers to help direct BC treatment. Prognostic markers can have implications for surveillance schedules and early intervention. When intervention is needed, predictive biomarkers can personalize treatment decisions by helping to identify those who would benefit most.

The European Association of Urology’s (EAU) 2022 guidelines note that evidence is still insufficient to routinely incorporate biomarkers such as molecular subtype or gene signatures into the clinical management of BC; however, several markers predictive of treatment response are acknowledged (5). The most recent version of the EUA guidelines, as well as those from the American Urological Association (AUA) and the National Comprehensive Cancer Network (NCCN) acknowledge particular circumstances in which the use of urinary biomarkers may be considered, indicating a growing body of evidence into the reliability of such markers (5–7).

**Figure 1:**
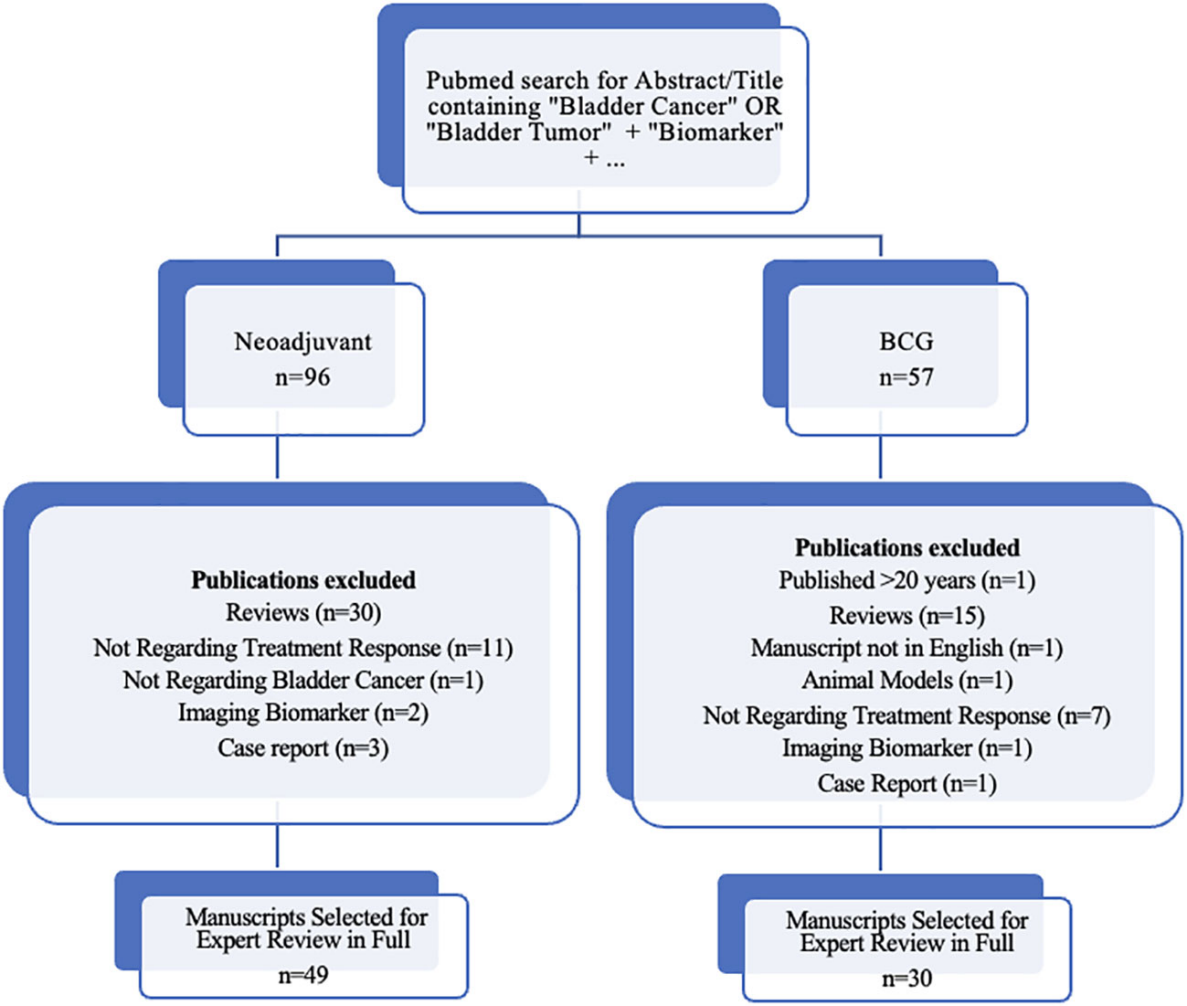
Diagram of search results and inclusion criteria.

In order to review the current evidence on promising new biomarkers, we performed a PubMed search using terms “biomarker,” and “bladder cancer,” or “bladder tumor,” and the respective treatment discussed (i.e., “neoadjuvant” or “BCG”), limited to terms appearing in the abstract or title to ensure that biomarkers in bladder cancer treatment was the primary topic of investigation. Articles published in the last 20 years were included in our initial review. **Figure 1** details our inclusion criteria for manuscripts selected for review in full. Articles describing novel biomarkers with high predictive potential were preferentially included, as well as validation studies of such biomarkers. We also included highly referenced systematic reviews where appropriate. In this article, we provide a review focusing on novel developments in predictive biomarkers in NMIBC and MIBC.

## 2 Non-muscle invasive bladder cancer

Approximately 75% of BC cases present as NMIBC, which includes superficial mucosal lesions (pTa), lamina propria invasion (pT1) and carcinoma *in-situ* (CIS) (2). The EAU guidelines risk-stratify patients with NMIBC into low, intermediate, or high risk based on the probability of disease progression (5). A combination of clinical and histopathological risk factors are used to determine a patient’s risk group. Currently, these are the only widely adopted predictive factors. In patients with intermediate and high-risk tumors, intravesical instillation of bacillus calmette guerin (BCG) after transurethral resection (TUR) reduces tumor recurrence and has been shown more effective than TUR and chemotherapy. Patients are maintained on a schedule of BCG instillation for 1-3 years depending on risk and provided that they demonstrate response. Despite treatment, 31-78% of patients will experience recurrence, and 1-40% will progress to muscle invasive disease at five years (8). The high rate of failure underscores the need for improved means of risk stratification.

Furthermore, 30% of patients experience some degree of systemic side effects, and many discontinue treatment (9). Therapy failure, tumor progression, and recurrence represent major challenges in the management of NMIBC. In cases which progress to MIBC without metastasis or lymph node invasion, cystectomy is the treatment of choice for appropriate surgical candidates. Evidence suggests that early cystectomy in high-risk NMIBC confers a cancer-specific survival advantage, while deferred cystectomy is associated with worse oncologic outcomes (10, 11). Biomarkers are needed to better identify patients at risk of recurrence or progression, and patients less likely to respond to BCG treatment.

### 2.1 Potential predictive biomarkers for BCG treatment

#### 2.1.1 Immune response and inflammatory markers

The mechanism of action of BCG is incompletely understood but is thought to involve local destruction of tumor cells as well as activation of the immune response and recruitment of inflammatory cells (12). Many steps in the pathway of immune response activation by BCG have been described. BCG attaches to fibronectin on the surface of urothelial cells before being internalized into the cell (13). Following internalization into the cell, BCG antigens are loaded onto major histocompatibility complex (MHC) II molecules and subsequently presented on the surface of urothelial cells where they are recognized by cluster of differentiation (CD) 4 cells, invoking at response by T helper-1 (Th-1) cells and inducing the release of interleukins (IL)-2, IL-12, interferon (IFN)- γ, and tumor necrosis factor (TNF)- β. This leads to recruitment of natural killer (NK) cells and cytotoxic T lymphocytes (CTLs) (14). These steps in the activation pathway point to potential immune and inflammatory biomarkers for predicting response to BCG. **Table 1** provides a summary of original studies in biomarkers predictive of BCG response or outcomes after BCG treatment.

**Table 1:** Biomarkers predictive of outcomes after BCG treatment.

Biomarker(s)	# Patients/Samples	Association with treatment response	Sens.	Spec.	PPV	NPV	AUC	Ref.
Immune Response and Inflammatory Markers								
105 cytokines, including: IL18BPa, IL23, IP10, IL8, SHBG, ITAC	50 - high/intermediate risk NMIBC	Increased % change IL18BPa and IL23 and Decreased % change IP10 and IL8 at 13 weeks –> worse FFS						(15)
IL-6/IL-10 ratio	72 high-risk NMIBC 49 controls - no malignancy	IL-6/IL-10 ratio > 0.10 associated with lower recurrence rate after BCG						(16)
IL-1β, IL-2, IL-6, IL-8. IL-10, IL-12, IFN-γ, TNF-α	20 - CIS	Higher levels of IL-2, IL-6, IL-8, IL-10, and TNF-α at the 8th BCG instillation associated with BCG response IL-2 level predictive of response in multivariable analysis						(17)
Oncuria™: APOE, A1AT, ANG, CA9, IL8, MMP9, MMP10, PAI1, SDC1 and VEGF	64 - high/intermediate risk NMIBC	Higher levels of CA9, ANG, and MMP10 prior to BCG associated with greater risk of recurrence 10-biomarker panel (Oncuria™) predictive of recurrence	81.8	84.9	0.529	0.957	0.897	(18)
NLR, PLR and LMR	125 - NMIBC (patients with only CIS excluded)	LMR > 3.25 predictive of progression after BCG					0.756*	(19)
Molecular Subtypes								
3 Novel BCG-response subtypes	132 - high risk NMIBC, BCG-naïve 44 - post-BCG recurrence	BCG-response subtype 3 associated with worse PFS, recurrence						(20)
3 Novel molecular subtypes	948 - NMIBC	DP.BCG+ subtype: worse PFS despite BCG response REC.BCG+ subtype: worse RFS despite BCG response						(21)

CIS, carcinoma in situ; LMR, lymphocyte-to-monocyte ratio; NLR, Neutrophil to Lymphocyte Ration; NPV, negative predictive value; PLR, platelet-to lymphocyte ratio; PFS, progression-free survival; PPV, positive predictive value; RFS, recurrence-free survival.

*in multivariable model including clinical and pathologic factors.

Because of their role as immune mediators, cytokines have been investigated as predictive biomarkers of BCG response. Urinary cytokine levels appear to increase during BCG treatment and are associated with survival outcomes and recurrence (22, 23). This implicates cytokines as potential non-invasive biomarkers, though further studies are needed to determine predictive combinations. In a study of 50 patients with high and intermediate risk NMIBC, Salmasi et al. measured urinary cytokine levels prior to BCG instillation and at interval time points throughout treatment. The authors found that the percent change of cytokines IL18BPa, IL23, IP10, and IL8 from baseline to 13 weeks was predictive of treatment response. Additionally, higher levels of urinary sex hormone binding globulin (SHBG) and lower urinary IFN-inducible T-cell α chemoattractant (ITAC) at 13 weeks predicted poor failure-free survival (15). Similarly, in a study of 20 patients with CIS, Watanabe et al. measured cytokine levels after the first and eighth instillation of BCG (17). Urinary IL-2 level at the later instillation was found to be independently associated with BCG response.

The ability to predict response early in the course of treatment is certainly important to distinguish those patients who are unlikely to benefit from further treatment. However, given that BCG treatment is not without side effects, biomarkers that can predict response prior to the first BCG instillation hold even greater clinical value. Cai et al. explored the pre-treatment ratio of IL-6, a T-cell growth and B-cell differentiation mediator, to IL-10 a known immune dysregulator, as a predictor of BCG response (16). The authors found that a higher (>0.10) IL-6/IL-10 ratio was associated with a lower recurrence rate. In a study of 64 patients with NMIBC, Murakami et al. tested the predictive ability of the commercially available urine-based diagnostic test Oncuria™ which measures 10 biomarkers associated with malignancy (18). The study found that pre-treatment levels of nine of these markers were associated with recurrence after completion of BCG treatment, with a sensitivity of 82% and specificity of 85%. These results have been subsequently validated in additional patients. This study suggests that contemporary investigation of biomarkers predictive of BCG response can take advantage of the clinical tests we already have. Indeed, Oncuria has recently gained FDA breakthrough designation as well as Conformite Europeenne (CE) marking in the EU (24). Based on these results, a multi-center phase 3 clinical trial of Oncuria as a predictive assay BCG treatment is underway (25).

UroVysion is another commercially available urine-based fluorescence *in situ* hybridization (FISH) assay with FDA approval for use in the diagnosis and surveillance of bladder cancer. Studies have evaluated the possibility of using a positive UroVysion analysis after the BCG induction cycle to predict poor response to BCG therapy and serve as an indicator of recurrence and progression (26). UroVysion has an advantage over singleplex protein-based markers and cytology due to its lower susceptibility to inflammatory influences and is therefore mentioned by the AUA guidelines as a possible tool to assess response to intravesical therapy (6). However, in a validation study in the same context, the test performance proved inadequate to guide decision making in an individual patient (27).

Several studies have described high neutrophil-to-lymphocyte ratio (NLR) as a prognostic factor for recurrence and progression in patients with NMIBC (28). A metanalysis by Vartolomei et al. examined six studies involving a total of 2,298 patients with various grades of NMIBC. Not only was high pre-treatment NLR found to be associated with worse recurrence-free survival, but in three studies, high NLR was also associated with decreased progression-free survival (PFS) in patients with high-risk NMBIC treated with BCG (29). Adamkiewicz et al. examined lymphocyte-to-monocyte ratio (LMR) in addition to NLR in patients undergoing BCG therapy and found LMR to be more predictive of progression after BCG (19). In summary, although urinary cytokine levels and peripheral immune cell ratios require further validation as prognostic markers, they represent potential for low-cost, non-invasive methods of determining response to BCG and selecting patients for early surgical intervention.

#### 2.1.2 Single gene/protein markers

Early studies focused on identifying a specific gene or gene product that was associated with disease outcomes. Typically these gene products had previously been implicated in the pathogenesis of other malignancies. Human epidermal growth factor receptor 2 (*HER2*), for instance, is an established prognostic factor in breast cancer. In comparing patients with low grade NMIBC, high grade (HG) NMIBC and papillary urothelial neoplasms of low malignant potential, Chen et al. found that *HER2* amplification in a subset of HG-NMIBC patients was associated with aggressive tumor behavior (30). In a study of patients with T1G3 NMIBC treated with transurethral resection of bladder tumor (TURBT) alone or TURBT + BCG, Cormio et al. found overexpression of *HER2* to be a stronger predictor of disease-free survival (DFS) and PFS than BCG treatment (31).

Fibroblast growth factor receptor (*FGFR*) alterations are seen in many types of cancer. *FGFR3* is involved in pathways of cell growth, cellular differentiation, and angiogenesis. Studies on the prognostic value of *FGFR3* in NMIBC have discordant findings. In a study of 80 patients with T1 disease, Sikic et al. found high expressions of *FGFR3* to be associated with increased risk of recurrence (HR=3.78) but improved overall survival (OS) (HR=0.50) (32). Hernandez et al. found no association between *FGFR3* and survival or recurrence in a sample of patients with T1G3 disease (33). Subsequent studies of FGFR3 as a single predictor (or in combination with another biomarker) of outcomes after BCG have failed to demonstrate statistical significance (34).

Although several early studies explored the potential of single prognostic and predictive biomarkers, results varied and large validation studies were lacking. Contemporary research has largely moved away from single biomarkers, favoring the discovery and validation of diagnostic signatures instead.

#### 2.1.3 Molecular subtypes and implications for NMIBC

Molecular subtypes of MIBC have been described and appear to affect response to therapy (35, 36). Similarly, recent studies classifying NMIBC into different molecular subtypes based on gene expression patterns have revealed implications for BCG response (37). De Jeong et al. performed molecular profiling of 132 BCG-naïve patients with high risk NMIBC and 44 patients with post-BCG recurrence and identified three distinct BCG response subtypes associated with differences in PFS and recurrence. The molecular subtype associated with the worst PFS and recurrence, termed BCG response subtype 3 (BRS3), was found to have overexpression of immunosuppressive genes, including PD-1/PD-L1 and chemokines, as well as overexpression of basal and epithelial-to-mesenchymal transition (EMT) activation markers. PFS in this cohort was 61%, compared to 78% and 83% in the other two molecular subtypes (20). Kim et al. similarly identified three molecular subtypes exhibiting distinct prognostic features (21). One subtype, termed DP.BCG+ showed worse PFS, even though the patients responded to BCG. Another subtype, REC.BCG+, showed worse recurrence-free survival (RFS) but also responded to BCG. Each subtype had at least three pathways whose activations were distinctly associated with that subtype. These studies provides further evidence that gene-based prognostic markers can be used to subclassify patients with NMIBC into prognostic and predictive subgroups, and also suggests specific pathways that may be implicated in progression and recurrence.

The development of massively parallel sequencing (or NGS) has dramatically changed our ability to detect genetic variations in malignant cells. Whereas traditional methods of genome and transcriptome analysis allowed for the interrogation of just a few candidate genes at a time, NGS has enabled genome-wide profiling as well as comprehensive transcriptome analysis including mRNA and non-coding RNA. Recently, next-generation sequencing (NGS) has been used to identify gene alterations in recurrent NMIBC that may correlate with response to BCG. Pietzak et al. performed NGS of 105 BCG-naïve patients with NMIBC. The authors identified alterations in *ERBB2* or *FGFR3* (mutually exclusive) present in the majority of high-grade tumors (57%) (38). Alterations in a chromatin-modifying gene, *ARID1A*, were significantly associated with an increased risk of recurrence after BCG [hazard ratio (HR) 3.14]. In a study using NGS of urine cytology from patients with high risk NMIBC, Scott et al. identified *TERT*, *TP53*, *ERBB2* and chromatin remodeling genes *KDM6A* and *ARID1A* as the most frequently altered genes (39).

Epigenetic changes have also been implicated in BCG response. Agundez et al. analyzed the methylation status of 25 tumor suppressor genes in patients with T1G3 NMIBC treated with non-maintenance BCG and found gene combinations predictive of disease progression (40). In a retrospective study of 108 patients with NMIBC, absence of methylation on *PMF-1*, a gene involved in cellular proliferation, was associated with recurrence HR: 2.03 and progression (HR:2.91) (41). Furthermore, in multivariable analysis, the authors found that among *PMF-1* methylation status and clinical and pathological factors of sex, age, focality, tumor size, and concomitant CIS, only *PMF-1* methylation was significantly associated with recurrence and progression. Epigenetic changes may therefore serve as a more precise means of risk-stratifying patients with NMIBC than the clinical and histopathologic features currently used.

There is clearly potential for existing and newly identified molecular subtypes to stratify patients based on response likelihood. Similar potential exists in using genetic alterations and epigenetic signatures as biomarkers in this space. However, follow-up studies in larger and more diverse cohorts are needed to further explore the clinical applicability of these markers.

## 3 Muscle-invasive bladder cancer

Approximately 25% of patients with BC present with muscle-invasive disease. Among those with NMIBC, 7-40% will experience progression at five years (8). For patients with primary or secondary MIBC, definitive treatment is with neoadjuvant systemic chemotherapy followed by complete resection. Neoadjuvant immunotherapy has been introduced in recent years, and serves as an alternative treatment for those ineligible for chemotherapy. Even with definitive treatment, patients with MIBC have high rates of recurrence and progression, and neoadjuvant therapies only benefit a small proportion of patients. Given the variability in clinical benefit, and availability of new treatment options, clinical biomarkers are needed to help direct treatment decisions. Here we review predictive biomarkers for response to systemic therapy in MIBC.

### 3.1 Neoadjuvant chemotherapy

Cisplatin-based neoadjuvant chemotherapy (NAC) followed by radical cystectomy (RC) with pelvic lymph node dissection is the standard of care for patients with MIBC. The primary roles of NAC are to reduce tumor size to enable complete resection and to treat micrometastases and improve the risk of recurrence (42). Appropriate regimens include methotrexate, vinblastine, doxorubicin, cisplatin (MVAC) and gemcitabine, cisplatin (GC) (43). Although both EUA and NCCN guidelines recommend NAC, this treatment is underutilized, with only 20% of patients with MIBC in the United States receiving NAC (7, 44). Low clinical application may in part be due to high rates of cisplatin ineligibility, particularly in an elderly population, and limited clinical benefit (45, 46). In patients with MIBC, NAC confers a 9% disease-free survival benefit and 5% overall survival benefit over 5 years, compared to local treatment alone (47). However, only approximately 40% of patients with MIBC show pathologic response to NAC (<pT1, N0 at time of cystectomy) and survival benefit appears to be limited to those with pathologic response (43). In patients who do not respond to systemic therapy, treatment with NAC unnecessarily delays surgery, which can negatively impact prognosis. Identification of those who will benefit from NAC is therefore imperative to reduce inappropriate treatment and overall morbidity and to expedite local treatment for patients unlikely to respond. Biomarkers for response to NAC have the potential to dramatically improve patient selection. While no biomarker has yet gained widespread clinical use, several promising developments have been made in predictive markers of response to NAC.

#### 3.1.1 New developments in molecular classification and their impact on MIBC treatment

There is substantial evidence to suggest that molecular differences among muscle-invasive bladder tumors impact response to treatment. Genomic profiling has dramatically improved our appreciation of the heterogeneity of BC and facilitated its classification into clinically meaningful molecular subtypes (48). Several molecular taxonomies for BC have been proposed over the years including a recent international consensus classification (**Table 2**) (49). The distinction between basal and luminal subtypes is recognized by all major bladder cancer taxonomies and their differing response to neoadjuvant treatment has been described in multiple studies (50). In a study of 269 patients receiving NAC, Seiler et al. reported that patients with luminal type tumors had the best overall survival regardless of NAC treatment, whereas patients with basal type tumors saw the greatest improvement with NAC compared to surgery alone (36). This differential benefit was later observed in a comparative study of 601 patients with MIBC, 40% of whom received NAC and 60% of whom underwent cystectomy alone. The authors found that non-luminal tumors had the highest benefit from NAC, with a 5-year overall survival benefit of 10%, compared to no significant survival benefit in those with luminal tumors (51). These data suggests that neoadjuvant chemotherapy may be most appropriate for patients with non-luminal BC subtypes.

**Table 2:** Classification systems for bladder tumor subtypes.

Classification (year)	# Subtypes	Subtypes described						
LUND (2012)	5	Urobasal A		Genomically unstable		Infiltrated	Urobasal B	SSC-Like
UNC (2014)	2	Luminal				Basal		
MDA (2014)	3	Luminal				Basal		p-53 like
TCGA (2017)	5	Luminal-Papillary	Luminal			Luminal Infiltrated	Squamous	Neuronal
Consensus (2020)	6	Luminal-Papillary	Luminal-Unstable		Luminal-non-specified	Stroma-Rich	Basal/Squamous	Neuroendocrine-like

NGS technology has allowed for a more granular classification system for BC. The Cancer Genome Atlas (TCGA; RRID : SCR_003193) project performed whole-exome and whole-genome sequencing on 131 bladder cancer specimens and later updated this sample to include a total of 412 patients (52, 53). Their analysis allowed for the stratification of bladder cancer into five mRNA-based subtypes: luminal-papillary, luminal-infiltrated, basal-squamous, luminal, and a previously undescribed neuronal subtype. Perhaps most exciting are the therapeutic implications of each subtype based on expression patterns. For instance, neuronal type tumors have high mutation rates in *TP52* and *RB1* genes, similar to other small cell neuroendocrine cancers (NEC); following the treatment pattern for other NECs, cisplatin-based therapy is suggested to be most appropriate for this subtype (54).

Gene expression profiling has yielded promising results toward a more personalized approach to MIBC treatment. For example, in a randomized Phase 2 trial (SWOG S1314), patients with MIBC were randomized to dose-dense (dd)MVAC or GC (55, 56). The primary goal of the trial was to assess the utility of the Co-expression Extrapolation (COXEN) algorithm, a treatment-specific gene-expression model, in predicting favorable response to therapy. COXEN scores were determined for each of the two treatments (COXEN GC and COXEN ddMVAC). The authors found that, while the treatment-specific scores were not predictive of response, the COXEN GC score was associated with pathologic downstaging in a pooled analysis of patients. More recently, in a secondary analysis, the authors found that the COXEN GC score was a significant predictor of overall survival in the pooled arm (abstract, presented at the 2022 American Society of Clinical Oncology) (57). Though further studies are needed, these findings highlight the potential of gene-expression profiling to discover predictive biomarkers and help direct treatment in this time of increasing therapeutic options for MIBC.

Furthermore, tumoral RNA from 303 MIBC treated with NAC were queried for 10 mRNA; *ANG, APOE, Serpine1, CA9, IL8, MMP9, MMP10, Serpina1, SDC1* and *VEGA*. This 10 gene panel (low expression *vs*. high expression of this signature) was able to predict recurrence free survival HR = 2.05 (1.19-3.52), p = 0.0118 (58).

In three studies, immunohistochemistry (IHC)-based or mRNA-based biomarkers were associated with differential outcome based on subtype (**Table 3**). Font et al. and Razzaghdoust et al. demonstrated an association between biomarkers of basal or basal/squamous subtype and improved pathologic complete response (pCR) (59, 60). However, a study by Jütte et all suggests that the luminal subtype is associated with improved pathologic response (61). These findings underscore a major limitation in the discovery of subtype-associated predictive markers thus far. Although some studies have been able to show a difference in response to NAC based on molecular subtype, others show no association (62). This discrepancy is likely due to various factors intrinsic to the studies themselves, including small cohorts and differing sequencing techniques. It has also been suggested that there exists significant intra-tumoral and intra-subtype heterogeneity which undercuts the prognostic value of subtyping. Gouin et al. described a previously uncharacterized subpopulation of MIBC cells distinguished by a high expression of N-Cadherin 2 and other epithelial markers (63). This subpopulation was seen in both luminal and basal subtypes and was associated with poor outcomes regardless of NAC, but improved outcomes with immune checkpoint therapy (ICT). Similar investigations are needed to improve predictions of therapy response based on subtype and to better substratify patients with MIBC.

**Table 3:** RNA and tissue based biomarkers of bladder cancer subtype.

Study, Year	n	NAC Regimen	Detection method	Marker and associated subtype	Predictive accuracy	Association of marker with outcomes
Font, 2020	126	GC, CMV, GCa	IHC	Low FOXA1/GATA3; High KRT5/6/14 –> Basal/Squamous High FOXA1/GATA3; Low KRT5/6/14 –> Luminal High FOXA1/GATA3; High KRT5/6; Low KRT14 –> mixed-cluster	NR	Basal/Squamous associated with higher chance of pCR (OR 3.96)
Jütte, 2021	54	GC	RT-qPCR	High expression KRT20, ERBB2, ESR1 –> Luminal	NR	Luminal associated with 40% higher chance of pCR
Razzaghdoust, 2021	63	GC, GCa	IHC	KRT5/6(+)/KRT20(-) –> Basal	NR	KRT5/6(+)/KRT20(-) (basal) associated with pCR

GC, gemcitabine and cisplatin; CMV, cisplatin, methotrexate, and vinblastine; GCa, gemcitabine and carboplatin; IHC, immunohistochemistry; NR, not reported; RT-qPCR, real time quantitative polymerase chain reaction; OR, odds ratio; pCR, pathologic complete response.

#### 3.1.2 Alterations in DNA repair pathways as predictors of response

Platinum-based therapies function by forming DNA crosslinks and interfering with cell division. DNA damage repair (DDR) mechanisms are elicited and, if unable to repair the damaged DNA, apoptosis is induced. MIBC is characterized by a high genetic mutational rate. Alterations in DDR genes have therefore been a substantial area of investigation in identifying which patients may benefit from cisplatin-based NAC. **Table 4** catalogues recent studies in DDR gene alterations and expression in predicting response to NAC.

**Table 4:** Alterations in DNA damage repair genes associated with response to NAC.

Gene alterations	Study, Year	n (NAC)	Detection method	Stage	Regimen	Definition PR	Path response outcome	Survival outcome
ATM, RB1, FANCC	Plimack, 2015	D-34 V-24	DNA sequencing	T2-4, N0-1, M0	ddGC, MVAC	≤pT1pN0cM0	Alteration in 1+ of the 3 DDR genes predicted PR and better OS in discovery and validation cohorts	
ERBB2 (HER2)	Groendijk, 2016	94	NGS	NR	GC, GCa, MVAC	<ypT2	*ERBB2* missense mutations associated with complete response	NR
ERCC2	Liu, 2016	55	WES	≥T2	GC, MVAC	<pT1	*ERCC2* alterations associated with response (OR 8.3; 95% CI, 1.4-91.4)	ERCC2 alterations associated with better OS
	Van Allen, 2014	58	WES	T2-T4, N0/N+, M0	GC, ddGC, ddMVAC, GC + sunitinib	pT0/pTis	*ERCC2* mutation associated with CR	NR
FGFR3	Yang, 2018	52	PCR, IHC	T0-4, N0/N+	GC	ypT1/pTa/pTis	*FGFR3* mutation associated with response	NR
7p12	Pichler, 2020	23	WES, IHC	T0-T4, N0-N3, M0	GC	ypT0-T1N0	7p12 amplification (genes *HUS1*, *EGFR*, *ABCA13*, and *IKZ*) associated with non-response (sens 71.4%, NPV 87.5%, spec 100%)	7p12

CI, confidence interval; D, discovery; dd, dose dense; GC, gemcitabine and cisplatin; GCa, gemcitabine and carboplatin; IHC, immunohistochemistry; MVAC, methotrexate, vinblastine, doxorubicin and cisplatin; NGS, next generation sequencing; NPV, negative predictive value; NR, not reported; OR, odds ratio; OS, overall survival; PR, pathologic response; PCR, polymerase chain reaction; V, validation; WES, whole exome sequencing.

DDR genes *ATM*, *RB1*, or *FANCC* have been implicated in the response of MIBC to platinum based NAC. In a 2015 study, Plimack et al. found that alterations in one or more of these genes was associated with pCR (64). A follow up study looking at long term outcomes (median follow-up 74 months) in this cohort showed that patients with alterations in at least one gene maintained better OS and DFS; the 5-year survival rate for patients with alteration(s) was 85% compared to 45% for those without (65).

*ERCC1* and *ERCC2* are nucleotide excision repair (NER) proteins that serve to repair damaged DNA strands. The correlation between increased expression of *ERCC1* and resistance to platinum-based chemotherapy has been well established across many types of cancer (66). Similarly early studies identified *ERCC1/2* as a prognostic marker in patients with MIBC. High expression of *ERCC1* is associated with worse disease-free survival and overall survival in patients receiving NAC (67). Recent studies demonstrate the association between genetic alterations to *ERCC2* and improved pathologic response after NAC (68, 69). Van Allen et al. performed whole exome sequencing of 50 patients with MIBC and found inactivating *ERCC2* mutations exclusively in responders to NAC. These results offer not only a predictive biomarker for response to NAC but also a targetable pathway to potentially improve cisplatin sensitivity (70).

*ERBB2* (aka *HER2*) is a receptor tyrosine kinases with potential for predicting response in MIBC. The association between *ERBB2* and poor prognosis in breast cancer is well established and led to the development of *ERBB2*-targeted therapies. *ERBB2* expression has also been associated with poor prognosis in bladder cancer (71). More recently, a study showed that *ERBB2* alterations are associated with pCR to treatment with NAC (72). *HER2* expression therefore offers a means of stratifying patients who are likely to benefit from platinum-based NAC.

#### 3.1.3 Non-coding RNAs

NGS allows for the interrogation of non-coding RNA (ncRNA) segments to assess for markers in these previously untested regions. The few recent studies on ncRNA as potential predictive biomarkers in MIBC reveal exciting new insight into our current classification system. A 2019 study by de Jong et al. identified a distinct subset of the mRNA-based luminal-papillary subtype using a long noncoding RNA (lnc-RNA) signature (73). This subgroup had favorable prognosis and was associated with distinct mutational profiles. Much like the Gouin study, de Jong’s analysis of ncRNA has the potential to further refine current molecular subtypes based on previously unidentified markers.

Discovery of biomarkers based on genetic alterations, tissue expression, and molecular subtype holds promise for selecting patients most likely to benefit from NAC. It is clear that the molecular characterization of MIBC into clinically relevant subtypes is still incomplete, though great strides have been made in the era of large-scale genomic sequencing. Currently, no single gene or protein-based biomarker has sufficient evidence to be incorporated into routine clinical practice when selecting patients for NAC, largely due to small sample sizes and the lack of validation studies.

### 3.2 Neoadjuvant immunotherapy

Although platinum-based NAC remains the standard of care, up to half of patients with advanced BC are ineligible for cisplatin (74). Treatment for ineligible patients or those who refuse pre-operative cisplatin has traditionally been upfront radical cystectomy. The recent approval of immune checkpoint inhibitors (ICI) for use in BC represents a breakthrough in treatment, as no new therapies had been approved for decades prior.

In 2016, the FDA approved programmed death-ligand 1 (PD-L1) inhibitor atezolizumab for the treatment of locally advanced and metastatic bladder cancer. Since then, four additional PD-L1 (durvalumab, avelumab) and PD-1 (nivolumab, pembrolizumab) inhibitors have been approved for use in bladder cancer. Based on their demonstrated efficacy and durable response in advanced and metastatic BC, PD-1/PD-L1 inhibitors are now being tested in the neoadjuvant setting (75). The ABACUS trial assessed the effectiveness of two cycles of atezolizumab as neoadjuvant therapy in a cohort of 88 patients with MIBC ineligible for cisplatin (76). The rate of pCR was 31% and the rate of downstaging was 39%. Higher response rates were achieved in the PURE-01 trial which assessed the efficacy of pembrolizumab in 50 patients with MIBC regardless of cisplatin eligibility (77). In this trial, the rate of pCR was 42% and the rate of downstaging to NMIBC was 54%. Trials assessing single agent and dual agent ICIs in the neoadjuvant setting have mostly yielded pCR rates similar to those seen with cisplatin-based therapies (78). As with NAC, a similar problem of limited response is encountered with ICIs (79). Therefore, a strong interest exists to identify biomarkers for stratifying response, and several of the randomized trials of ICIs have reported differential response based on immune-related markers.

Biomarkers such as PD-L1 expression, tumor mutational burden (TMB), and alterations in DDR genes have been associated with response to PD-L1 inhibitors in patients with metastatic BC (80, 81). A 2018 study of 50 patients from the PURE-01 trial found that high pre-operative TMB, DDR alterations, and PD-L1 expression independently correlated with pCR (77). The association between TMB and PD-L1 expression was upheld in a subsequent study of the updated PURE-01 cohort which included patients with variant histology (82). In this study, the association was present regardless of histologic subtype, suggesting these markers may be a better predictor of response to ICI than histopathologic features. Powels et al. found no correlation between these markers and outcomes of pathologic response or recurrence-free survival in patients enrolled in the ABACUS trial (76, 80).

The contradictory findings highlight a limitation in the assessment of biomarker-based response to ICI thus far. Expression assays and cutoff values vary by study, rendering pooled or comparative analyses challenging. For instance, biomarker analysis from the ABACUS trial analyzed the expression of PD-L1 on immune cells using a 5% cutoff. By comparison, studies of the PURE-01 cohort used the combined positive score (CPS) which includes PD-L1 expression on tumor and immune cells.

Biomarker analysis in the ABACUS trial did find that a high presence of intraepithelial CD8+ cells preoperatively was significantly associated with improved pCR (40%, compared to 20% in those with absent CD8). Also predictive was an 8-gene cytotoxic T-cell transcriptional signature (tGE8), a marker of interferon signalizing that indicates the presence of CD8+ effector cells. It has been established across multiple cancers that immune infiltration, that is, the proximity of CD8+ cells to the tumor, may influence response to PD-1/PD-L1 therapy even more than effector cell density (83). The immune infiltration of tumors is classified into three phenotypes with demonstrated predictive value for ICI response (84). The inflamed phenotype is characterized by infiltration of CD8+ and CD4+ T cells into the parenchyma of the tumor; in the excluded phenotype, immune cells are present but remain in the stroma surrounding the cancer cells; finally, the desert phenotype describes an absence of abundant immune cells in both the parenchyma and the stroma. Unsurprisingly, tumors with inflamed phenotype have been associated with better response to ICIs (83, 85).

Accordingly, biomarkers that classify patients based on immune phenotype and expression of effector cells may be a powerful way to distinguish ICI responders. As in the case of NAC, advances in genomics and transcriptomics have facilitated the identification of potential phenotype-based markers. Jiang et al. constructed nine-gene signature that was found to correlated with level of immune infiltration (86). This signature was able to predict immunotherapeutic response in two cohorts with an AUC of 0.64 and 0.69 respectively. You et al. identified two genes, discoidin domain receptor (DDR) 1 and 2, whose mutually exclusive expression patterns correlated with immune phenotype in BC. High expression of DDR2 indicated a T-cell inflamed phenotype whereas high levels of DDR1 indicated a non-inflamed phenotype. Furthermore, DDR signature score was developed which predicted overall survival in two independent cohorts undergoing ICI therapy (87). These studies offer a potential way to stratify ICI responders based on multi-gene expression signatures.

## 4 Circulating tumor cells and extracellular vesicles

Beyond the genetic and surface protein expression of tumor cells themselves, the contents released by tumors have recently been studied as potential biomarkers in both NMIBC and MIBC. Circulating tumor cells (CTCs) are malignant cells in the peripheral blood derived from the primary tumor. These disseminated cells have the potential to evolve clinically significant distant metastases (88). The detection of high levels of CTCs in serum may therefore hold prognostic potential. A large metanalysis comprising 20 studies and 1,339 patients found that CTCs were detectable in 20-30% of patients with NMIBC compared to 40-100% of patients with MIBC (89). A recent studies demonstrated that the number of CTCs is significantly higher in patients with MIBC compared to those with NMIBC and higher in those with high-grade compared to low grade BC, suggesting a potential role in risk-stratifying patients (90). CTCs have even been studied in the context of prognosis after RC and response to NAC. In a study of 100 patients with advanced non-metastatic BC undergoing RC, the detection of CTCs in the peripheral blood preoperatively was associated with significantly higher risk of recurrence, cancer-specific mortality, and overall mortality (91). Yang et al, among a total cohort of 196 patients, measured pre-treatment CTCs in 32 NMIBC patients undergoing NAC followed by RC (92). They found that tetraploid CTCs were able to distinguish NAC-sensitive patients from NAC-resistance patients with an AUC of 0.80. Although studies are mostly limited by small sample sizes, CTCs represent an exciting potential for minimally invasive biomarker analysis as they do not require sampling of the primary tumor.

Similarly, there has been a growing interest in the diagnostic and prognostic potential of extracellular vesicles (EVs) in BC. EVs are packages of genetic information and proteins released by virtually every type of cell, including tumor cells, and mediate cell-cell communication. The role of tumor EVs in promoting cancer progression has been established in multiple cancers, including BC (93, 94). The lipid bilayer of EVs stabilize their contents from degradation. Serum and urinary EVs are therefore being investigated as a way to diagnose BC or distinguish patients at higher risk of progression. Studies have focused on the concentration of urinary EVs as well as their contents. One study found significantly higher concentration of EVs in patients with bladder cancer which could distinguish them from healthy controls with a sensitivity of 81% and specificity of 90% (95).

Due to the presence of EVs in the urine and ongoing efforts to find urinary biomarkers to outperform cytology, many of the studies on EVs have centered on their diagnostic potential. However, recent studies have alluded to their prognostic value as well. Zhan et al. evaluated the expression of eight lnc-RNAs in urinary EVs (96). The authors found that increased expression of two lnc-RNAs, PCAT-1 and MALAT, was associated with poor RFS.

## 5 Conclusion

The treatment landscape of bladder cancer is quickly changing with the introduction of immunotherapy. Improved sequencing techniques have dramatically contributed to our understanding of BC as a heterogenous disease and enabled the development of several biomarkers with the potential to help predict response to treatment and appropriately select patients for these. In the case of NMIBC and MIBC, no biomarker has yet been adopted in routine clinical practice; however, the use of biomarker-based patient selection in recent clinical trials promises to refine our understanding of how certain markers drive response. The majority of biomarkers in bladder cancer have not yet transitioned from bench to bedside. However, it is hopeful that soon one or several compelling predictive biomarkers will emerge and be incorporated into standards of care.

## Author contributions

PC, DT, CR, and MA contributed to conception and structure of the manuscript. PC wrote the first draft of the manuscript. DT, CR, and MA wrote sections of the manuscript. All authors contributed to manuscript revision, read, and approved the submitted version.
